# Qualities of an Effective Clinical Teaching Fellow in Medical Education—A Mixed‐Methods Study

**DOI:** 10.1111/tct.70516

**Published:** 2026-08-31

**Authors:** Rocco Sheldon, Andrew Gan, Thomas Fleck, Richard Phillips, Elizabeth Peterknecht

**Affiliations:** ^1^ College of Medicine and Health University of Birmingham Birmingham UK; ^2^ Manchester University NHS Foundation Trust Manchester UK; ^3^ University Hospitals Birmingham NHS Foundation Trust Birmingham UK

**Keywords:** clinical teaching fellow, cross‐sectional study, educational workforce, medical education, skills, teaching development, teaching effectiveness

## Abstract

**Introduction:**

Clinical teaching fellows (CTFs) are an increasingly important part of undergraduate medical education in the United Kingdom. Despite their valuable and uniquely multifaceted role, research investigating the qualities that make an effective CTF is limited. This study aimed to identify and compare the views of stakeholder groups regarding the most important qualities of CTFs.

**Methods:**

We conducted a single‐centre mixed‐methods study at a large UK teaching hospital. An online questionnaire was distributed to clinical medical students (years 3–5), CTFs, administrative staff and faculty. Data were collected via Likert scales, asking respondents to rank qualities and teaching experiences and free‐text responses.

**Results:**

Quantitative analysis (*n* = 43) identified friendliness, being knowledgeable and an engaging teaching style as the most valued qualities. Prior experience of conducting bedside teaching and small‐group tutorials was prioritised, with postgraduate teaching qualifications and specialist knowledge ranked as least important. Medical students valued professionalism and teamwork significantly less than other groups (*p* < 0.05). Qualitative thematic analysis revealed students prioritised a supporting learning environment and practical clinical tips. Conversely, administrative staff, faculty and CTFs valued collaborative teamwork and organisational reliability. Reflecting these differences, 29 (67.4%) respondents agreed with our hypothesis that disparities exist between stakeholder groups in the qualities/experiences they value most.

**Conclusions:**

This study highlights commonalities and differences regarding the interpersonal skills and teaching experiences that are central to effective CTFs. These findings have important implications for recruitment, training and professional development of CTFs, ensuring alignment with the needs of stakeholders and addressing perceived disparities between groups.

## 1 Introduction

Clinical teaching fellows (CTFs) are doctors, typically in the early postgraduate years, appointed to roles with protected time for teaching. These posts were introduced in the United Kingdom in the 1990s to enhance undergraduate teaching provision and to offer doctors structured opportunities to develop as educators [1]. Although the title ‘CTF’ is UK‐specific, similar posts exist internationally, such as chief residents in North America and structured clinical educator pathways in Australasia, indicating the broader relevance of this model [2, 3].

Posts are generally offered for a minimum of 12 months, with time either dedicated entirely to teaching, or split between clinical work and educational/professional development. The delivery of undergraduate teaching sessions includes case‐based discussions and plenaries, simulation, communication and procedural skills, as well as clinical examination tutorials [4]. However, these posts also require the assumption of many other roles aside from that of the teacher, such as leader, mentor, innovator, examiner and researcher [5].

CTFs are uniquely placed relative to other clinical educators due to these widespread responsibilities and deep integration in the organisation and delivery of the undergraduate medical curriculum. However, no research to date has explored the qualities and attributes that make an effective CTF across multiple stakeholder groups in a clinical setting. Formal quantitative analysis is also limited, which may supplement findings from existing qualitative studies. This mixed‐methods study seeks to fill this gap by exploring the opinions expressed by four distinct stakeholders in undergraduate education within an NHS trust setting—medical students, education faculty members, current CTFs and administrative staff in the education department. We also aim to analyse the differences in opinion between the groups and why these differences may exist. Extrapolating disparities identified between stakeholders in preclinical CTF roles [6], we hypothesise that there is a significant difference in what medical students value in a CTF versus other stakeholder groups.

Additionally, despite their numerous benefits, many challenges relating to the CTF role have been documented [7, 8]. This underscores the need for evidence‐based frameworks to guide their recruitment and training. We hope that the findings from our study will be used to ensure the most appropriate applicants are appointed to these highly influential roles, support discussions aimed at bridging stakeholder perspectives and to ultimately improve teaching quality and student satisfaction.

## 2 Methods

### 2.1 Design, Ethics and Study Setting

This was a mixed‐methods explorative study undertaken using surveying to explore key stakeholder insights into the characteristics that make an effective CTF. The quantitative component was designed to identify differences in stakeholder perceptions, with a qualitative component to explore underlying explanations. Participants were all based at Sandwell and West Birmingham Hospitals (SWBH) NHS Trust. Consent was obtained at the start of the survey and participant responses were anonymised. Ethical approval was sought from SWBH R&D. All ethical considerations pertinent to this study are fully elucidated in the protocol, available upon request.

### 2.2 Study Population

We targeted distinct populations including medical students in years 3–5 of the undergraduate MBChB programme, current/previous CTFs based at SWBH, and members of the undergraduate academy and administrative team at the trust. A range of population groups was selected to represent all relevant stakeholders involved in undergraduate education at SWBH from August 2020 to August 2022. The full inclusion and exclusion criteria are outlined in Table S1.

### 2.3 Methods of Data Collection

Participants who met the inclusion criteria and provided informed consent were asked to complete ‘The Good CTF’ questionnaire. The questionnaire was locally developed by two former CTFs (T.F. and E.P.) studying postgraduate medical education and iteratively refined. Based on prior experience, their reflexive stance is that enthusiasm, reliability and knowledge equivalent to a second‐year postgraduate doctor are the most important qualities of a CTF. The questionnaire was reviewed and approved by two senior clinical educators with no changes before obtaining ethical approval. Content validity was established using expert review, consistent with Lynn's framework for quantifying content validity in instrument development [9]. Although no prior validation studies exist, items were informed by the clinical teaching literature and aligned with established domains of teacher effectiveness [10].

The questionnaire was created on Survey Monkey (SurveyMonkey Inc., San Mateo, California, USA.) and distributed via email to all eligible participants (Table S2). Targeted recruitment numbers were 120 medical students, 8 CTFs and 10 undergraduate admin/faculty members at SWBH. Power calculations demonstrated that 91, 7 and 9 respondents in each respective group were required for 90% power.

### 2.4 Analysis

#### 2.4.1 Quantitative Analysis

Responses to Questions 2–14 were imported into SPSS for analysis. Descriptive statistics were used to calculate the proportions of respondents from each group. Box plots were generated based on the responses to the 7‐point Likert scales for Questions 3–10. For Question 11, the ranked responses for each aspect of teaching experience were used to generate box plots. For Questions 12 and 13, box plots were generated based on ranking the ordinal data from least to most experience. Answers to Question 16 were coded as ‘Agree’ or ‘Disagree’ to generate a stacked bar chart.

Kruskal–Wallis tests were performed for Questions 3–13 to investigate differences between responder groups. For Questions 14 and 16, Fisher's exact tests were performed to investigate differences in responses by stakeholder groups.

#### 2.4.2 Qualitative Analysis

Open‐ended survey responses to Questions 15 and 16 were analysed thematically following the step‐wise approach [11], guided by frameworks of clinical teaching effectiveness. All quotes and their corresponding participant number and role were imported into Microsoft Word. Quotes were divided by responder group and read thoroughly by investigators to achieve immersion. The comment function of Microsoft Word was used by R.S. and A.G. to independently code responses, with disagreements resolved by discussion. Themes were developed collaboratively between the two investigators. The coders were fourth‐year medical students at the SWBH after the data collection period. Consequently, their perspectives as learners may have attuned them to themes of near‐peer empathy and supportive environments. To mitigate against potential bias, independent coding was performed, with themes reviewed by the wider team consisting of three former CTFs. No artificial intelligence was used to analyse results. Our analysis should be understood as exploratory thematic coding, consistent with guidance on the limitations of open‐ended survey data [12].

## 3 Results

### 3.1 Survey Participants

Of the 138 people the survey was sent to, 43 participants completed the survey (response rate of 31.2%). Of which, 14 (32.6%) were third year medical students, 4 (9.3%) were fourth year medical students, 11 (25.6%) were fifth year medical students, 7 (16.3%) were CTFs, 4 (9.3%) were undergraduate administrative staff and 3 (7%) were undergraduate faculty members (Table S3).

### 3.2 Quantitative Results

#### 3.2.1 Importance of CTF Qualities

**FIGURE 1:**
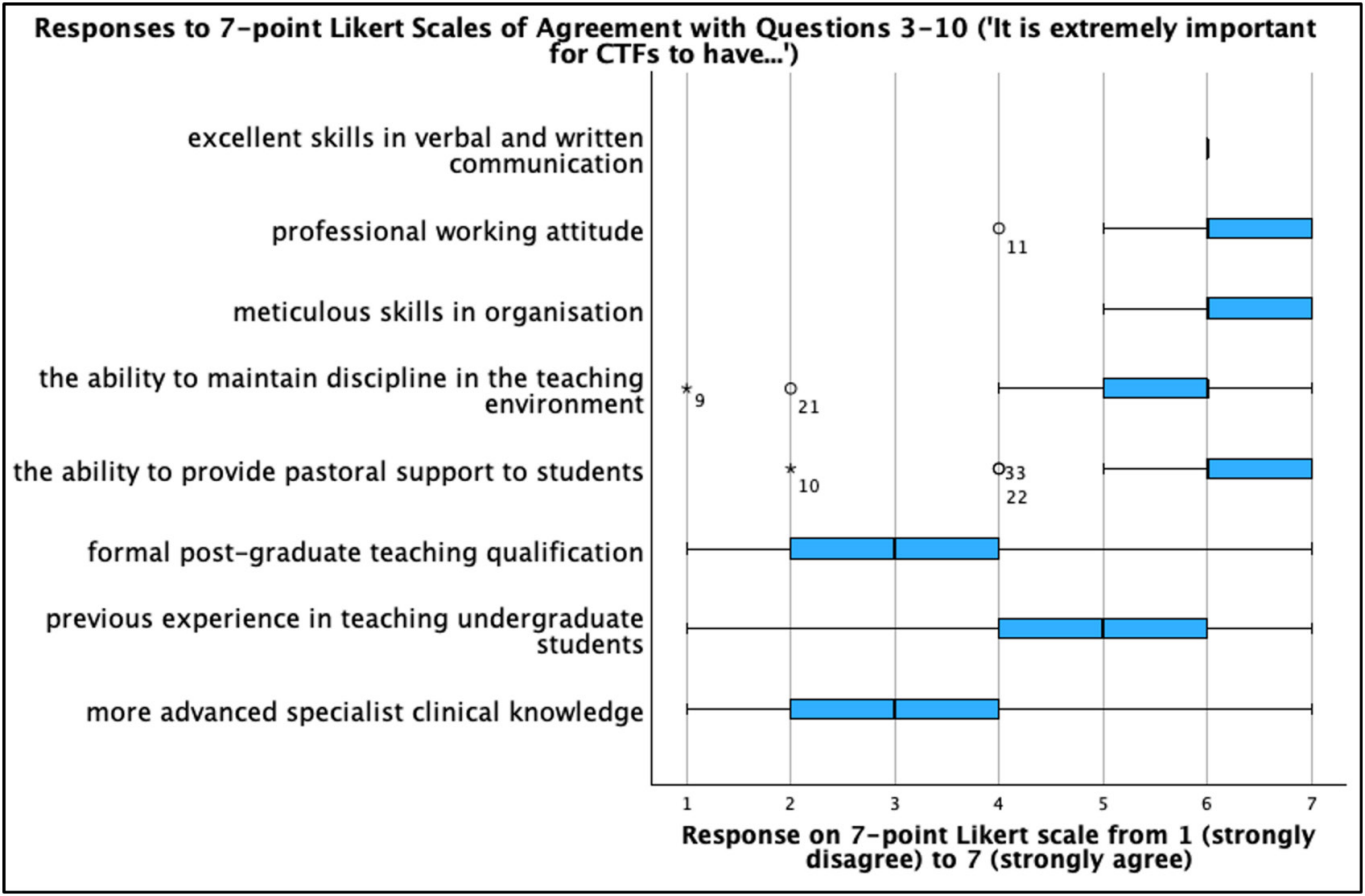
Box plot for Questions 3–10 regarding the importance of a variety of qualities CTFs may have on a 7‐point Likert scale.

Descriptive statistics were calculated for responses to Questions 3–10 regarding the importance of CTFs to have each aspect of prior experience. Box plot responses to the 7‐point Likert scales used for these are shown in Figure 1.

The median response for Questions 3–7 was ‘Agree’, indicating broad consensus about the importance of communication skills, a professional working attitude, organisational skills, the ability to maintain discipline and provision of pastoral support.

The median response to Question 9 regarding the importance of previous teaching experience was ‘Somewhat agree’, but with great variation between ‘Strongly disagree’ and ‘Strongly Agree’. The same variation was also seen in Questions 8 and 10 regarding CTFs needing prior teaching qualifications and specialist knowledge, with a median response of ‘Somewhat disagree’.

We then conducted subgroup analysis by responder group to investigate the causes of this variation. Box plots were produced illustrating variation between groups; however, these did not reach statistical significance (Figures S1–S3).

#### 3.2.2 Importance of Prior Teaching Experience

In Question 11, participants were asked to rank which teaching modalities from most (1) to least (6) useful for a CTF to have past teaching experience in. Modalities included delivering lectures, delivering small‐group tutorials, delivering simulation, delivering bedside teaching, one‐to‐one tuition, delivering clinical skills teaching.

**FIGURE 2:**
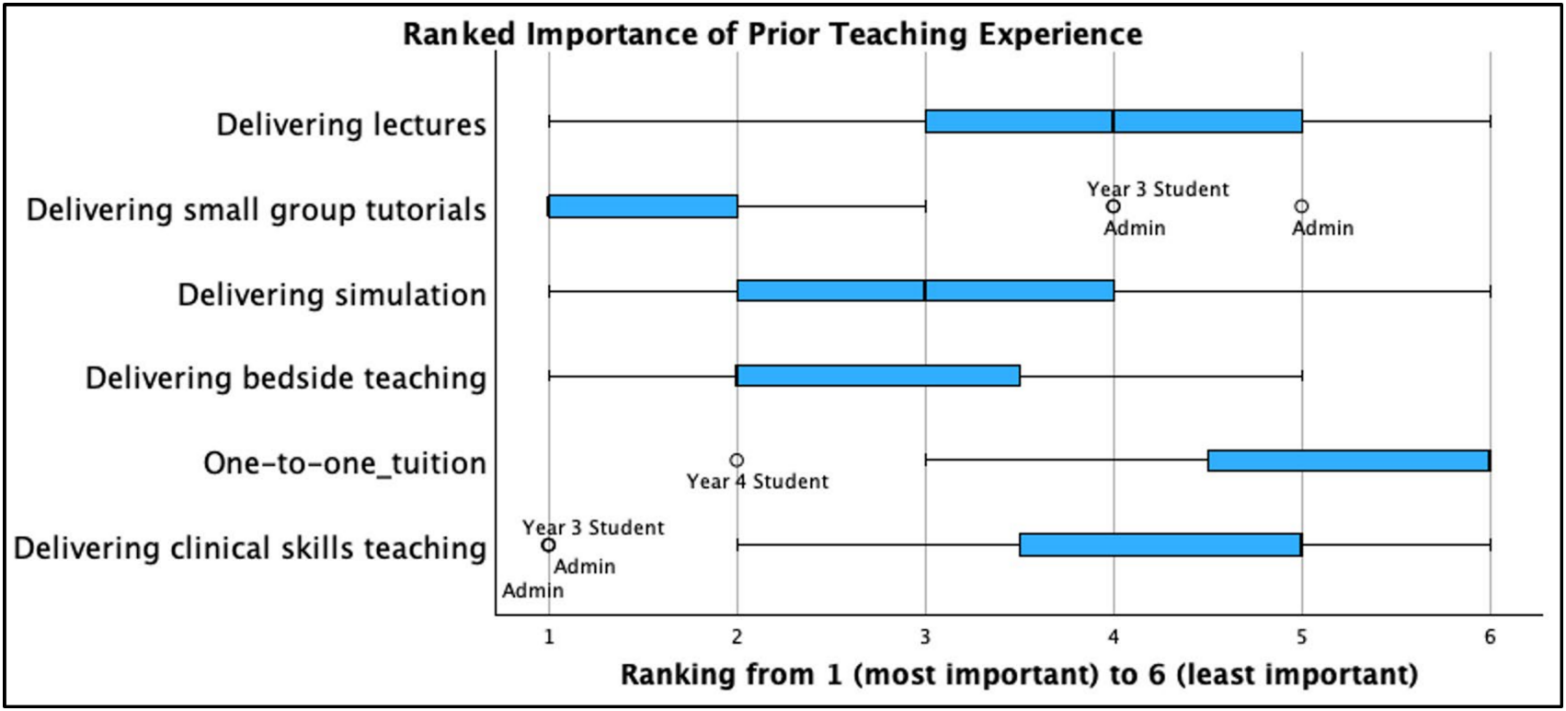
Box plot illustrating the ranked importance of prior teaching experience in the following areas: delivering lectures, small group tutorials, simulation, bedside teaching, one‐to‐one tuition and clinical skills teaching. Outliers ranking and their responder groups are annotated.

Overall, experience delivering small groups tutorials was valued significantly more than other experiences (*p* < 0.001), whereas experience of one‐to‐one tuition (*p* < 0.001) and delivering clinical skills teaching (*p* < 0.001) was valued significantly less than experiences (Figure 2).

Box plots for median ranking of each aspect by position of respondent were performed to investigate causes for variation between respondents (Figures S4–S7).

Kruskal–Wallis tests were performed to assess differences between groups. These indicated a statistically significant difference between groups in the ranking of ‘delivering bedside teaching’ only (*p* = 0.036), driven by third‐year medical students placing particular emphasis on it.

Statistical significance was also approached in the ranking of ‘delivery of small group tutorials’ (*p* = 0.059), ‘delivery of simulations’ (*p* = 0.079) and ‘delivery of clinical skills teaching’ (*p* = 0.147). Small group tutorials were valued less by admin than all other groups, whereas simulations were valued more by admin and faculty than all other groups. Prior experience of clinical skills teaching was similarly valued highly only by members of the undergraduate admin team.

Questions 12 and 13 aimed to establish how much teaching experience participants thought CTFs should have, and how much they have in practice. The median response for both questions was 1–4 weeks. There were no significant differences between the amount of teaching experience respondents thought CTFs should have (Question 12) and how much they have in practice (Question 13) (Figure S8).

**FIGURE 3:**
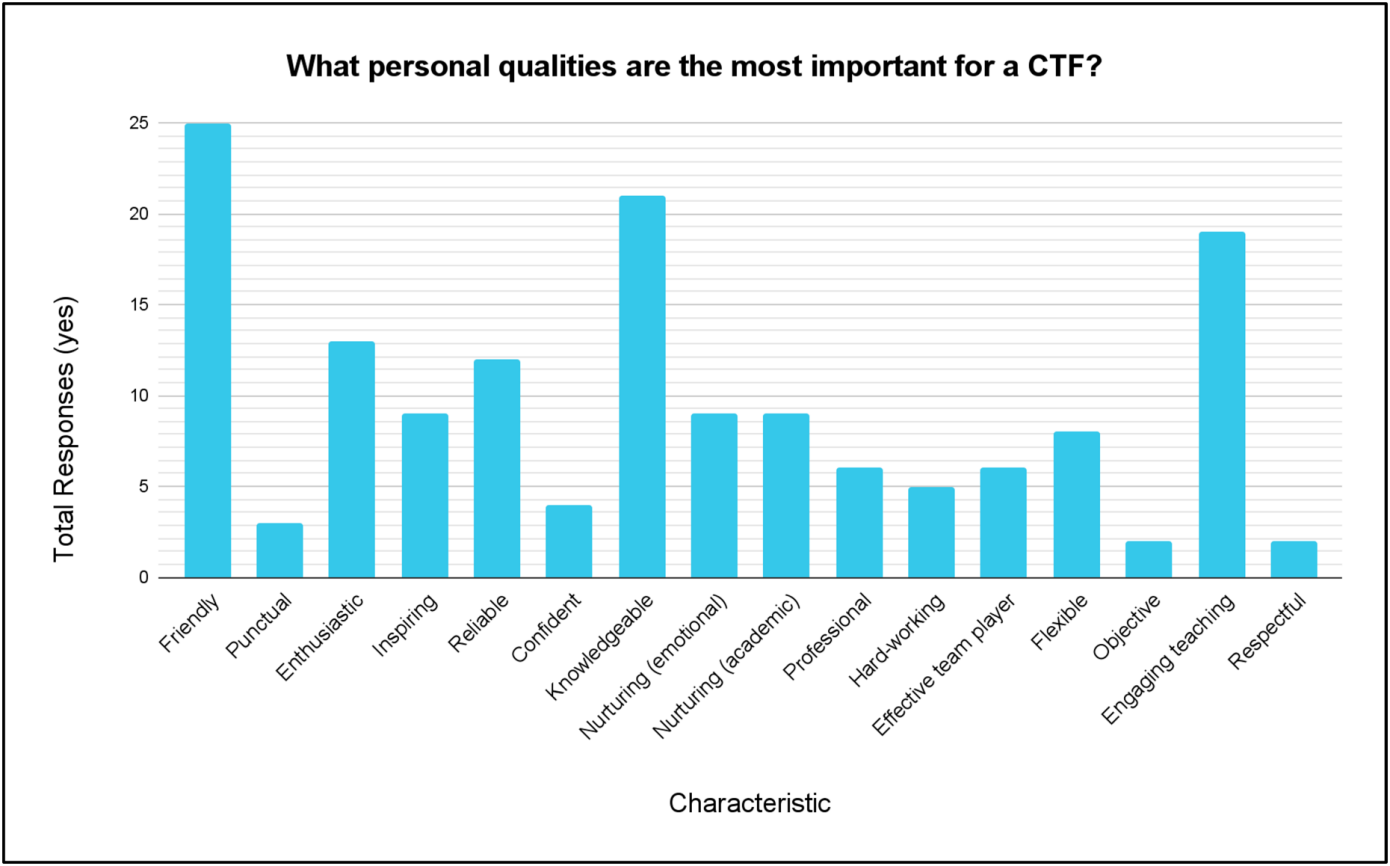
Total number of respondents who ranked each of the predetermined characteristics in the top 3 most important for CTFs to have.

Question 14 asked participants to rank the top three qualities they believe are most important for CTFs to have from a predefined list (Figure 3). The most identified qualities were ‘friendly’ (*n* = 25), ‘knowledgeable’ (*n* = 21) and ‘engaging teaching style’ (*n* = 19).

Fisher's exact tests were performed for each characteristic to determine if there was an association between the responder group and their responses. The *p* value for each characteristic is presented in Table S4.

Five characteristics had statistically significant association with the responder group, as highlighted in the table. Notably, ‘professional’, ‘hard‐working’ and ‘effective team player’ were stated more than expected by admin, faculty and CTFs when compared with responses from medical students (*p* < 0.05).

### 3.3 Qualitative Analysis

#### 3.3.1 What Qualities Make a Good CTF?

**FIGURE 4:**
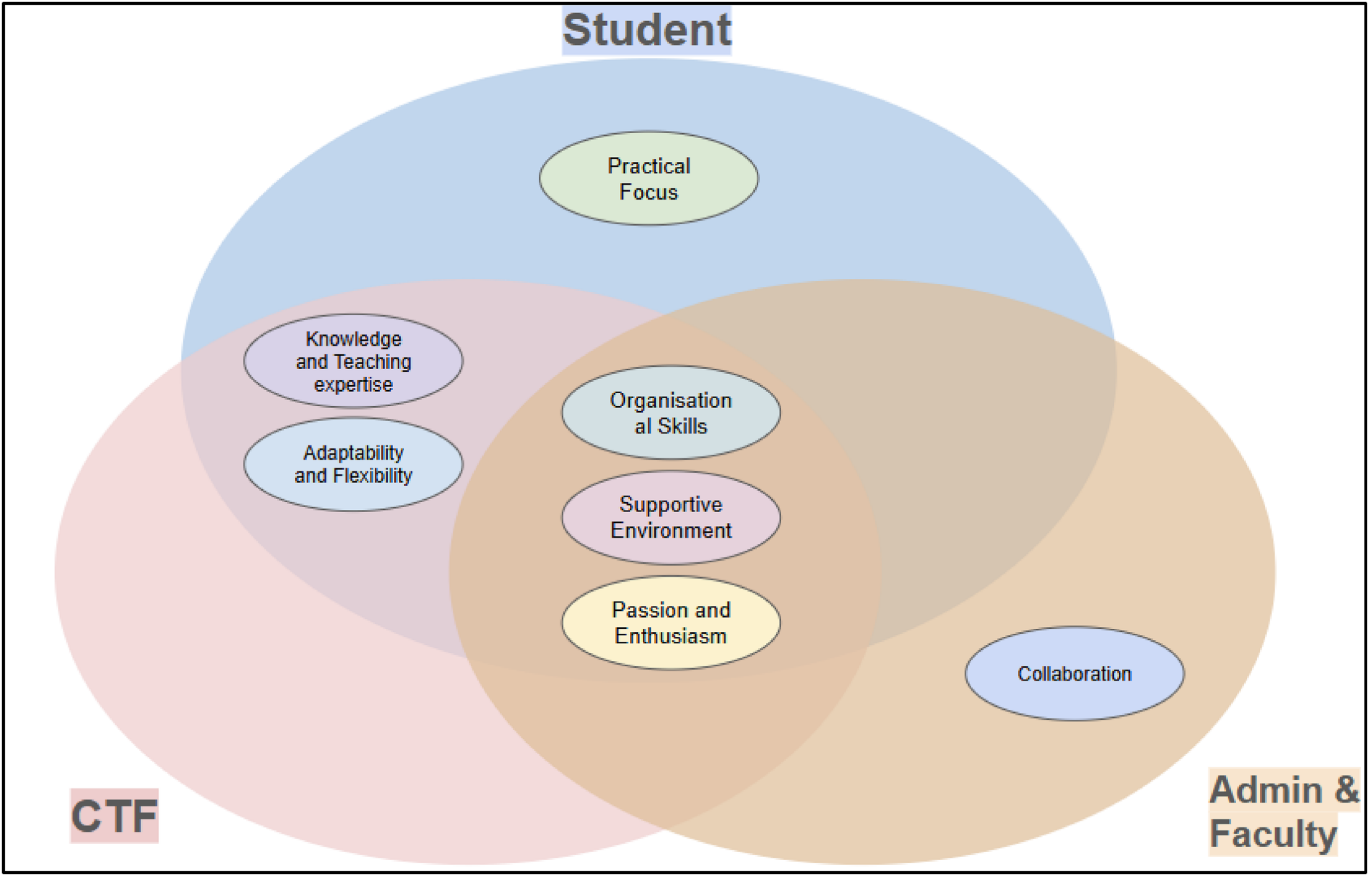
Venn diagram illustrating the distribution of themes identified in Question 15 by responder group.

In total, 43 participants responded to Question 15. Thematic analysis was used to analyse the results as previously described. Final themes were concretely defined and linked to their subthemes before being organised into a Venn diagram (Figure 4). Appendix S1 contains a narrative report and verbatim quotes.

#### 3.3.2 Is There a Disparity in the Qualities That Stakeholder Groups Perceive Make a Good CTF?

Question 16 stated our hypothesis that there is a disparity in the qualities that medical students, CTFs, admin and faculty members perceive make an effective CTF. Of the 35 valid responses, 29 (82.9%) agreed that a disparity existed compared with 6 (17.1%) who disagreed. No differences in agreement rates were found between respondent groups (*p* = 0.356) (Figure S9).

Of these 29 respondents who agreed with the hypothesis, 17 (58.6%) stated the disparity lay between students and admin/faculty members, 6 (20.7%) between CTFs and admin/faculty and 2 (6.9%) between students and CTFs.

**FIGURE 5:**
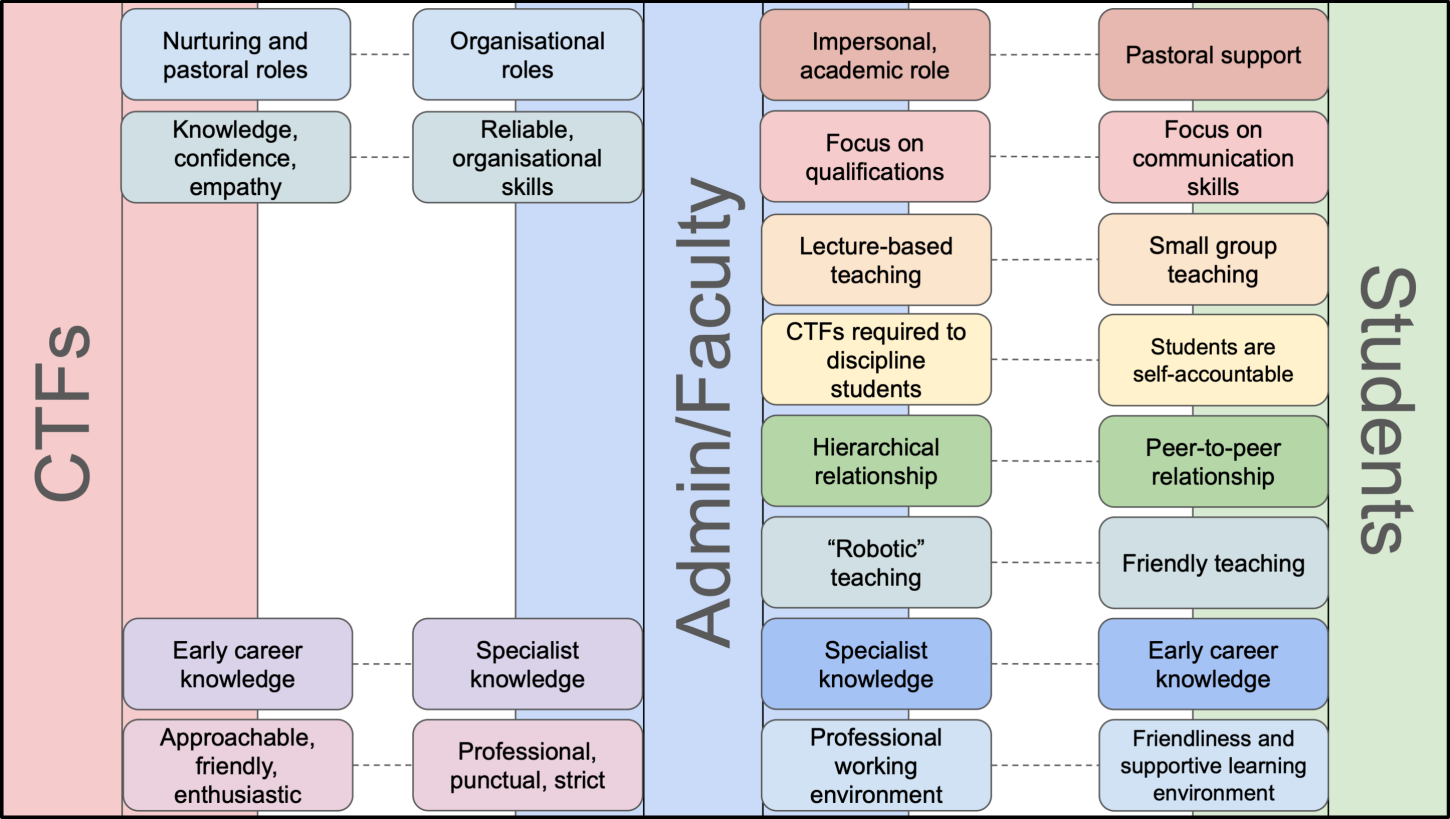
Diagram illustrating the responses of students and CTFs to Question 16 and their perceived disparities between themselves and admin/faculty members. In their respective columns, the perceived priorities of CTFs and students are given, linked to what they believe admin/faculty members prioritise instead. Similar themes identified between CTFs and students have been placed in the same horizontal row.

Qualitative analysis of Question 16 was limited to students and CTFs due to the paucity of responses from administrative staff and faculty members. Figure 5 details the perceived disparities identified by students and CTFs between themselves and admin/faculty members. Detailed analysis is presented in Appendix S2.

## 4 Discussion

Our analysis identified the salient qualities consistently associated with effective CTFs by stakeholders. These findings corroborate existing research on the characteristics of high‐quality clinical instruction [10, 13, 14, 15, 16].

From a practical perspective, institutions can leverage these themes to inform recruitment criteria, interview processes and induction programmes for new CTFs. Emphasising structured development opportunities, mentorship from senior educators and ongoing formal training further ensure that CTFs maintain currency and confidence in their teaching roles [14, 17].

### 4.1 Core Attributes of an Effective CTF

A consistently engaging teaching style represented a unifying theme. This resonates with prior research that effective CTFs motivate and involve students in bedside tutorials through well‐structured teaching methodologies and clearly defined objectives [14]. Furthermor students particularly value the reliable and engaging teaching that CTFs provide relative to other clinical teachers [18]. Furthermore, the concept of ‘engagement’ aligns with broader pedagogical literature on active learning, which posits that strategies such as small‐group discussions and scenario‐based teaching can enhance knowledge retention and clinical reasoning skills [13].

All respondents highlighted the importance of knowledgeable CTFs possessing a thorough understanding of the medical school curriculum, aligning with prior research [6, 19, 20, 21]. Consequently, prospective CTFs who have graduated more recently and from the same university may be best positioned to bridge the divide between theoretical knowledge and the practicalities of clinical care [17].

Finally, participants stressed the importance of reliability, indicating that CTFs play a vital role in modelling professional behaviours. Administrative staff particularly valued reliability in organising sessions, which has been recognised in the literature to maintain student trust and enhance the overall learning environment [14].

### 4.2 Differences in Perceptions Between Groups

Notable differences were found between responder groups regarding the qualities that make an effective CTF (Figure 4). Several complementary theoretical frameworks help to contextualise these differences.

One such framework is role theory, whereby stakeholders' expectations and priorities are driven by their position within the educational system [22]. For example, collaboration, professionalism and teamwork were valued more highly by admin/faculty members and CTFs, whereas medical students, as sole recipients of teaching, neglected this domain. This pattern is consistent with existing preclinical research [6].

An area of agreement across all stakeholder groups was the importance of approachability in CTFs. However, only students identified the importance of practically focussed teaching and a supportive learning environment, the latter perhaps reflecting the inherently vulnerable and emotionally challenging role of students on clinical placement [23]. These two uniquely student‐reported preferences are supported by the social and cognitive congruence of CTFs valued by many students: sharing similar life experiences knowledge frameworks may permit more effective teaching in a ‘psychologically safe’ environment relative to more senior clinicians who are further removed from learners in age, experience and teaching style [15, 24, 25].

Within Lave and Wenger's situated learning theory, medical education represents a journey from ‘legitimate peripheral participation’ as a student observer to core membership in a clinical community [26]. CTFs span the boundary between these two positions, which may explain why their qualitative responses both empathise with students and recognise the organisational priorities of the undergraduate faculty.

*Only students identified the importance of practically focussed teaching and a supportive learning environment*.

The results of Question 16 were stark: particularly students believing, or assuming, a disparity between themselves and ‘the system’ (faculty and administrative staff). Student experiences of burnout, mistreatment and unprofessional behaviour may represent violations of the psychological contract between students and their training institutions, driving mistrust [27]. This may be further amplified by the rise in ‘student consumerism’ and expectations of ‘value‐for‐money’ from training programmes [28]. Greater transparency in recruitment process and communication between stakeholder groups to highlight the many areas of agreement identified by this study may increase cohesion and satisfaction with CTF roles.

*Students believing, or assuming, a disparity between themselves and ‘the system’ (faculty and administrative staff)*.

### 4.3 The Role of Teaching Experience and Qualifications

It is clear all responder groups value a knowledgeable teacher; however, most disagreed that prior teaching qualifications are necessary for the role of a CTF. This indifference aligns with prior research which found that only 21% of students believed formal teaching qualifications are required to deliver the best teaching, and 43.5% were unaware of whether their clinical teachers possessed these qualifications [18]. Additionally, CTFs tend to complete a postgraduate certificate or diploma during their year(s) as a CTF, and therefore a prior qualification is uncommon.

*Most disagreed that prior teaching qualifications are necessary for the role of a CTF*.

Experience with teaching modalities such as bedside teaching and small group tutorials was consistently valued highly in our study, with students emphasising these approaches as crucial for understanding clinical skills and fostering engagement. This aligns with evidence from Brown [19], which underscores the benefits of interactive and hands‐on teaching experiences.

*Experience with teaching modalities such as bedside teaching and small group tutorials was consistently valued highly*.

Conversely, one‐to‐one tuition was consistently ranked as the least important teaching modality for prior experience. This contrasts with qualitative responses from students emphasising the importance of their personal relationships with CTFs and the transfer of employability and relevant skills inherent to near‐peer teaching [29]. This may be explained by students assuming that these skills are an integral part of being an early‐career doctor, thus highlighting the unique ability early‐career CTFs have to empathise with students [30].

The implications for CTF selection and training are clear regarding the most prioritised teaching experiences. Integrating experiences from bedside teaching, small group tutorials and simulation equips prospective CTFs with the practical skills needed to address diverse learning needs. Additionally, as seen in the ripple effect study by van Heerden et al. [15], these experiences can contribute to a more cohesive teaching environment within institutions, ultimately benefiting both educators and learners by fostering a dynamic, student‐centred approach to clinical education.

## 5 Limitations

Although the present study provides valuable insights into the stakeholder perspectives on what makes an effective CTF, several limitations must be acknowledged.

One notable limitation of this study is the small sample size, with a response rate of only 31.16% (43 out of 138 participants). This relatively low response rate—particularly from CTFs (*n* = 7), administrative staff (*n* = 4) and faculty (*n* = 3)—limits the generalisability of the findings, as the views expressed by participants may not fully reflect broader populations. Statistical comparisons between groups may also lack sufficient power (set at 0.9) to detect meaningful differences, potentially leading to Type II errors (false negatives). Furthermore, there is the potential for varied interpretations of questions inherent to research surveys and the study is subject to potential response bias, as those who chose to participate may have had stronger opinions or experiences regarding CTFs.

Another limitation of the study is its single‐site design. Teaching environments, institutional structures and expectations for CTFs can vary significantly between institutions, which may influence the relevance of our results to other settings. Reproducing this study at different institutions is necessary to determine whether the findings are generalisable to a wider population of medical schools in the United Kingdom and among similar clinical educator roles globally. Additionally, not collecting the views of physicians, allied health professionals and patients represents a blind spot to be addressed by future research.

This reliance of questions on recall introduces the possibility of recall bias, as participants may have difficulty accurately remembering past experiences or may focus on particularly positive or negative events, skewing their responses. The reliance on open‐ended questions limited qualitative insights into explanations of heterogeneous stakeholder perceptions; the use of focus groups and interviews in future studies may ameliorate this. Additionally, this study relied entirely on participants' perceptions of what constitutes an effective CTF, which may not correspond with actual CTF effectiveness; measuring objective outcomes such as student exam performance or faculty evaluations will provide more comprehensive insights into this.

## 6 Conclusion

There is broad agreement on the importance of communication, professionalism, organisation and enthusiasm for a CTF. Practical and interactive teaching approaches (small‐group and bedside teaching) are seen as the most valuable experiences for CTFs. Students value approachability, practical teaching and engagement, whereas faculty and admin prioritise professionalism, teamwork and reliability. There are some perception gaps between students, CTFs, faculty and admin regarding the importance of specialist knowledge, prior teaching experience and formal teaching qualifications. Particularly, the high rates of students believing disparities exist between themselves and those organising their teaching require greater collaboration to address. Although CTFs are UK‐based, our findings provide insight into how to optimise the design, recruitment and training for early‐career clinical teaching posts internationally.

*Students value approachability, practical teaching and engagement, whereas faculty and admin prioritise professionalism, teamwork and reliability*.

Future research should be conducted across a greater number of sites with different CTF roles and in different areas of the country. CTFs are often supported by simulation fellows and clinical skills educators in delivering undergraduate teaching, and this practice varies between trusts. The roles of these allied education professionals should also be explored to identify how these roles can be best placed to deliver teaching effectively and in accordance with their specialisations.

This remains the first study investigating the qualities that constitute a ‘good CTF’ across a variety of stakeholders in a clinical setting. We have identified a broad agreement in effective qualities, and the integration of these into the recruitment and training of CTFs will improve the experience of students, CTFs, and administrative and faculty staff alike.

## Author Contributions

**Richard Phillips:** conceptualization, methodology, supervision, writing – original draft, writing – review and editing, investigation. **Andrew Gan:** writing – original draft, writing – review and editing, visualization, formal analysis, data curation. **Elizabeth Peterknecht:** writing – original draft, writing – review and editing, conceptualization, methodology, supervision, investigation. **Thomas Fleck:** conceptualization, methodology, writing – original draft, writing – review and editing, supervision, investigation. **Rocco Sheldon:** writing – original draft, writing – review and editing, visualization, formal analysis, data curation.

## Funding

No specific grant was received for the research or authorship of this article. Open access publication charges were supported by the University of Birmingham under its institutional open access agreement.

## Conflicts of Interest

The authors declare no conflicts of interest.

## Supporting information


**Table S1:** Participant inclusion and exclusion criteria.
**Table S2:** Questions and response options for the questionnaire administered via surveymonkey.com.
**Table S3:** Participant group characteristics.
**Table S4:** Total number of respondents who ranked each of the predetermined characteristics as in the top three most important for CTFs to have.
**Figure S1:** Box plot of responses to Question 8 (‘It is extremely important for a CTF to have a formal post‐graduate teaching qualification (e.g., PG Cert, PGDip, Masters, PhD) before starting work as a CTF’) from each group along a 7‐point Likert scale.
**Figure S2:** Box plot of responses to Question 9 (‘It is extremely important for a CTF to have previous experience in teaching undergraduate students before starting work as a CTF’) from each group along a 7‐point Likert scale.
**Figure S3:** Box plot of responses to Question 10 (‘Assuming current CTFs have at least FY2 level clinical knowledge, it is extremely important for a CTF to have more advanced specialist clinical knowledge’) from each stakeholder group along a 7‐point Likert scale.
**Figure S4:** Box plot illustrating the ranked importance of prior teaching experience in delivering small group tutorials by stakeholder group.
**Figure S5:** Box plot illustrating the ranked importance of prior teaching experience in delivering simulation by stakeholder group.
**Figure S6:** Box plot illustrating the ranked importance of prior teaching experience in delivering bedside teaching by stakeholder group.
**Figure S7:** Box plot illustrating the ranked importance of prior teaching experience in delivering clinical skills teaching by stakeholder group.
**Figure S8:** Box plot illustrating the desired prior teaching experience CTFs should have according to each stakeholder group.
**Figure S9:** Box plot illustrating the rates of agreement and disagreement to Question 16 by stakeholder group.

## Data Availability

The data that support the findings of this study are available from the corresponding author upon reasonable request.
