# Supplementary material for: Qualities of an Effective Clinical Teaching Fellow in Medical Education—A Mixed‐Methods Study
Source: Clin Teach. 2026 Aug 31;23(5):e70516. doi: 10.1111/tct.70516 (PMC13529404; doi:10.1111/tct.70516)
Supplement: Supplementary file 1 [file TCT-23-e70516-s001.docx]

Supplementary Material: Qualities of an effective clinical teaching fellow in medical education – a cross-sectional study

*Supplementary Table 1. Participant inclusion and exclusion criteria.*

| **Inclusion Criteria** | **Exclusion Criteria** |
| --- | --- |
| - Medical students from the University of Birmingham and Aston Medical School, in their 3rd, 4th or 5th academic year of the undergraduate MBChB programmes. - Clinical teaching fellows employed by Sandwell and West Birmingham Hospitals NHS Trust (SWBH), from the period of August 2020 – August 2022, to deliver undergraduate teaching. - Academic members of the undergraduate academy at SWBH involved in appointing CTFs to post and/or frequent interactions with the CTFs. - Members of the undergraduate administrative team at SWBH involved in appointing CTFs to post and/or frequent interactions with the CTFs. - Provided appropriate consent | - CTFs currently at trusts other than SWBH are not included in this study. - Undergraduate faculty members from other trusts and from the respective universities are not included in this study. - No exclusion of participants based on gender, age, ethnicity, socio-economic grouping or location. - Any participant who would not consent for themselves were excluded from this study. - Any participant who did not meet the inclusion criteria were excluded from this study. |

*Supplementary Table 2: Questions and response options for the questionnaire administered via surveymonkey.com.*

| **Question Number** | **Question** | **Answer** |
| --- | --- | --- |
| 1 | Do you consent to participate? | Yes / No |
| 2 | What group are you in? | - 3rd / 4th / 5th year medical student - Clinical Teaching Fellow - Undergraduate Administrative staff - Undergraduate Faculty |
| 3 | It is extremely important for a CTF to have excellent skills in verbal and written communication. | 7-point Likert scale  (from 1 - strongly disagree to  7 - strongly agree) |
| 4 | It is extremely important for a CTF to have a professional working attitude. | 7-point Likert scale  (from 1 - strongly disagree to  7 - strongly agree) |
| 5 | It is extremely important for a CTF to have meticulous skills in organisation. | 7-point Likert scale  (from 1 - strongly disagree to  7 - strongly agree) |
| 6 | It is extremely important for a CTF to have the ability to maintain discipline in the teaching environment. | 7-point Likert scale  (from 1 - strongly disagree to  7 - strongly agree) |
| 7 | It is extremely important for a CTF to be able to provide pastoral support to students. | 7-point Likert scale  (from 1 - strongly disagree to  7 - strongly agree) |
| 8 | It is extremely important for a CTF to have a formal post-graduate teaching qualification (e.g. PG Cert, PGDip, Masters, PhD) before starting work as a CTF. | 7-point Likert scale  (from 1 - strongly disagree to  7 - strongly agree) |
| 9 | It is extremely important for a CTF to have previous experience in teaching undergraduate students before starting work as a CTF. | 7-point Likert scale  (from 1 - strongly disagree to  7 - strongly agree) |
| 10 | Assuming current CTFs have at least FY2 level clinical knowledge, it is extremely important for a CTF to have more advanced specialist clinical knowledge (i.e. knowledge expected of a trainee who has already commenced training in a specific speciality e.g. elderly care medicine, general surgery etc.). | 7-point Likert scale  (from 1 - strongly disagree to  7 - strongly agree) |
| 11 | What previous teaching experience is most important in your opinion? Please rank the following items from most important (1) to least important (6) | Delivering:   - Lectures - Small group tutorials - Simulation - Bedside teaching - One-to-one tuition - Clinical skills teaching |
| 12 | How many hours/days/weeks etc. of actual teaching experience do you think are desirable for a CTF before starting the post (on average)? | - None - <1 day - 1-7 days - 1 week - 4 weeks - 1 month - 6 months - >6 months |
| 13 | Before starting the CTF post, how many hours/days/weeks etc. of actual teaching experience do you think a CTF in-post has actually completed (on average)? | - None - <1 day - 1-7 days - 1 week - 4 weeks - 1 month - 6 months - >6 months |
| 14 | What personal qualities are the MOST important for a CTF? (Tick ONLY 3 BOXES) | - Friendly - Punctual - Enthusiastic - Inspiring - Reliable - Confident - Knowledgeable - Nurturing (emotional) - Nurturing (academic) - Professional - Hard-working - Effective team player - Flexible - Objective - Engaging teaching style - Respectful |
| 15 | ‘Please think of a CTF who you thought was effective in their role. What reasons can you provide that made them particularly effective in post? Please provide as much detail as possible (without naming the person).’ | Free text |
| 16 | ‘Our hypothesis is that there is a disparity between what medical students, CTFs, undergraduate faculty and administrative staff believe are qualities that make an effective CTF. Do you agree with this statement, and why?’ | Free text |

*Supplementary Table 3. Participant group characteristics.*

| **Category** | **n** | **%** |
| --- | --- | --- |
| 3^rd^ Year Medical students | 14 | 33 |
| 4^th^ Year Medical students | 4 | 9 |
| 5^th^ Year Medical students | 11 | 26 |
| CTFs | 7 | 16 |
| Faculty members | 3 | 7 |
| Administrative staff | 4 | 9 |
| **Total** | **43** | **100** |

*Supplementary Table 4. Total number of respondents who ranked each of the predetermined characteristics as in the top three most important for CTFs to have.*

| **Characteristic** | **Total Responses (yes)** | **P-value** |
| --- | --- | --- |
| Friendly | 25 | 0.331 |
| Knowledgeable | 21 | 0.604 |
| Engaging teaching style | 19 | 0.058 |
| **Enthusiastic** | **13** | **0.009** |
| Reliable | 12 | 0.662 |
| Nurturing (emotional) | 9 | 0.372 |
| Nurturing (academic) | 9 | 0.783 |
| Inspiring | 9 | 0.118 |
| Flexible | 8 | 0.079 |
| **Professional** | **6** | **0.024** |
| **Effective team player** | **6** | **0.046** |
| **Hard-working** | **5** | **0.020** |
| Confident | 4 | 0.692 |
| Punctual | 3 | 0.277 |
| **Objective** | **2** | **0.043** |
| Respectful | 2 | 0.206 |

P value refers to differences in rankings between responder groups. Statistical significance was defined as p<0.05 and highlighted in bold.

*Supplementary Figure 1. Box plot of responses to question 8 (“It is extremely important for a CTF to have a formal post-graduate teaching qualification (e.g. PG Cert, PGDip, Masters, PhD) before starting work as a CTF”) from each group along a 7-point Likert scale.*

*Supplementary Figure 2. Box plot of responses to question 9 (“It is extremely important for a CTF to have previous experience in teaching undergraduate students before starting work as a CTF”) from each group along a 7-point Likert scale.*

*Supplementary Figure 3. Box plot of responses to question 10 (“Assuming current CTFs have at least FY2 level clinical knowledge, it is extremely important for a CTF to have more advanced specialist clinical knowledge”) from each stakeholder group along a 7-point Likert scale.*

*Supplementary Figure 4. Box plot illustrating the ranked importance of prior teaching experience in delivering small group tutorials by stakeholder group.*

*Supplementary Figure 5. Box plot illustrating the ranked importance of prior teaching experience in delivering simulation by stakeholder group.*

*Supplementary Figure 6. Box plot illustrating the ranked importance of prior teaching experience in delivering bedside teaching by stakeholder group.*

*Supplementary Figure 7. Box plot illustrating the ranked importance of prior teaching experience in delivering clinical skills teaching by stakeholder group.*

*Supplementary Figure 8. Box plot illustrating the desired prior teaching experience CTFs should have according to each stakeholder group.*

*Supplementary Figure 9. Box plot illustrating the rates of agreement and disagreement to question 16 by stakeholder group.*

# Supplementary Appendix S1: Qualitative analysis of Question 15

**Theme 1: Creating a supportive environment**

This theme encompasses many subthemes, such as being approachable, providing friendly, non-judgemental support in learning. Students also elaborated on the importance of empathy and mentorship in CTFs, potentially aided by their common recent experiences of medical student life.

*“...they made the group teaching environment a friendly place so I felt comfortable enough to ask questions. This is so so important, because then I was not afraid to ask and therefore could learn what I didn't know without being made to feel stupid.” - Year 3 medical student, responder 39.*

*“They were able to sense when a student is disheartened and provide support and encouragement … "believe in yourself, you can do it".” - Year 3 medical student, responder 33.*

Mentorship was particularly noted by a fifth-year student as they approach working life as an FY1 doctor.

*“Approachable for questions about academics, future training, or for pastoral support.” - Year 5 medical student, responder 29.*

Administrative and CTF views on this theme were limited, but they acknowledged the utility of being responsive to questions.

*“They were approachable and helpful whenever I had queries” - Admin, responder 15*

Notably, when applying a frequentist approach to the qualitative data set, creating a supportive environment was the most mentioned theme, noted by 11 respondents. Triangulating this data with the earlier results from question 14, which identifies “friendly” as the most important CTF trait, shows the approachability of a CTF is clearly crucial.

**Theme 2: Passion and Enthusiasm**

Passion about teaching was noted by all respondent groups as a positive quality in a CTF. This passion manifested as enthusiasm in the delivery of teaching.

*“Enthusiastic to teach and clearly wanted us to learn and enjoyed teaching us.” - Year 5 medical student, respondent 41.*

Linked to this passion was a hard-working attitude and distinct motivation to improve, which were both particularly valued by admin and faculty staff.

*“This individual was hard working” - Admin, respondent 15*

*“They were driven and had lateral thinking” - Faculty, respondent 32*

Specifically, the detrimental effects of a lack of passion on enjoyment of teaching sessions was stated by one student.

*“[The] best CTFs are the ones that actually show they care about the students' education and improvement. It's very obvious when CTFs are there just for the pay cheque. Also if a CTF starts every session with "this isn't my area of expertise" then it gets pretty annoying especially when their job is to teach us the content.” - Year 5 medical student, respondent 27.*

This theme is further validated by the results from question 14, which identified “enthusiastic” in the top 3 most important CTF traits.

A subtheme of ‘Passion and Enthusiasm’, noted only and simultaneously most commonly by students, is its effects on engagement and therefore learning of students.

This subtheme has a strong link to creating a supportive environment, and this inclusive teaching is in turn maintained by students to encourage participation and hence build confidence. Another key facilitator of student engagement is noted to be active learning styles rather than passive lectures, with constantly asking questions and drawing diagrams to explain concepts noted to be particularly effective.

*“They were very kind and supportive. They believed in all of us and voiced praise where it was due. They corrected our mistakes without making us feel silly.” - Year 4, respondent 6*

*“Their teaching style was SO engaging - they would ask us questions as they taught the topic. And instead of delivering lectures which are so passive, they would draw out diagrams of what we were learning which was SO much more effective. It really helped me learn and remember topics so much more” - Year 3, respondent 39*

Despite being the joint most cited quality by medical students at 9 responses, this subtheme was not mentioned by any other responder group.

**Theme 3: Organisational Skills**

Reference to planning and organisation featured heavily within the free text answers of all responders. Students and CTFs focussed on reliability and proactiveness in the organisation of teaching, particularly bedside teaching sessions.

*“Being organised and ahead of the calendar in terms of organising teaching sessions/materials.” CTF, respondent 21*

*“Made the effort to ensure we always had bedside teaching planned once a week” - Year 3, respondent 33*

A key aspect of organisation and planning of teaching is communication with colleagues to ensure sessions run smoothly. This was acknowledged by fellow CTFs, and admin staff as the two key stakeholders in the organisation of teaching.

*“Organised and able to communicate well with other CTFs to plan and deliver sessions.” - CTF, respondent 16*

*“Always kept colleagues in the loop and informed” - Admin, respondent 23*

**Theme 4: Knowledge and Teaching Expertise**

Awareness of relevant subject matter and how to deliver it was a widely cited quality amongst students and CTFs alike but not referenced by admin staff or faculty members.

Strong subject knowledge was deemed to be a desired quality by both groups. This includes an awareness of the university curriculum, assessment, and what their own knowledge gaps were.

*“Confident in knowledge (including gaps in knowledge)” - CTF, respondent 43*

*“They knew what we had to learn for our curriculum and focused our teaching on that.” - Year 4, respondent 6*

*“Regular schedule of teaching that comprehensively covered the entire content of the block” - Year 3, respondent 5*

Effective teaching techniques were also a focus of students and CTFs alike. Systematic, focussed teaching was valued, as well as accommodating teaching to meet the needs of students.

*“They kept the lectures concise and to the point in regards to clinical knowledge we have to know, displayed in a systematic and step-by-step way; thus, making it easy to follow” - Year 3, respondent 20*

*“They listened to student feedback and organised teaching sessions relevant to what we wanted to learn.” - Year 5, respondent 3*

A culture of feedback is an essential part of clinical teaching. This feedback is bidirectional and is greatly valued by students for giving them personalised advice to improve their skills.

*“There was also good correction and teaching specific to each individual in terms of each of our flaws in history taking and performing examinations.” - Year 3, respondent 33*

**Theme 5: Adaptability and Flexibility**

The importance of CTFs adapting their teaching style to meet students’ needs was recognised by students and CTFs alike, but not by administrative staff or faculty members.

*“He came to every session very prepared but was also very flexible in allowing to change the subject or mode of teaching (e.g changing bedside teaching to tutorial teaching).” - Year 3, respondent 25*

*“Being dynamic in teaching styles to accommodate for all learning types.” - CTF, respondent 21*

Flexibility was also noted as important by both CTFs and students, but for differing reasons. CTFs valued flexibility in terms of covering teaching sessions at short notice, whereas students valued accommodating for the teaching requests of students, and the stress students are under before exams.

*“They were flexible and understood the stress that I was under as a final year student and accommodated for me during teaching” - Year 5, respondent 10*

*“They listened to student feedback and organised teaching sessions relevant to what we wanted to learn.” - Year 5, respondent 3*

**Theme 6: Collaboration**

‘Collaboration’ encompassed two main aspects, teamwork and effective communication, which facilitate collaborative session planning. Teamwork was particularly mentioned by admin staff, who identified qualities such as friendliness and being supportive as key parts of teamwork.

*“They are a team player and have always been polite and there to support the whole team.” - Admin, respondent 15*

Checking understanding between team members about teaching plans was also deemed desirable by a member of admin staff. Admin and faculty shared a common desire for CTFs being “reachable” for any queries and having “excellent communication”.

*“Willing to meet as a team to make sure that the teaching plan was understood by all members of the team” - Admin, respondent 23*

Notably, this collaborative theme was mentioned only by admin and faculty and not by the students who are the recipients of the CTF teaching.

**Theme 7: Practical Focus**

‘Practical focus’ was a theme only mentioned by medical students. These students particularly enjoyed bedside teaching and using these cases to discuss the investigations and management for these conditions.

*“Bedside teaching was really good, it was very structured. Someone took a history, someone did the exam whilst someone had to think about their investigations and management. Then there was discussion-based teaching and we looked at imaging and investigations done on the patient we saw” - Year 3, respondent 17*

One student noted that a useful part of this is practical clinical tips gained from the CTF’s own experience. A fifth-year student elaborated on these practical tips in terms of advice to prepare them for being an FY1 doctor.

*“Provided teaching on things that you can’t learn yourself, eg skills on being a good f1” - Year 5, respondent 36*

# Supplementary Appendix S2: Qualitative analysis of Question 16

**Students vs Admin/faculty**

In general, students believed that admin/faculty members value a professional relationship between CTFs and students, and therefore place much less emphasis on the importance of friendliness and approachability. Students maintained that this hierarchical relationship is not optimal for learning, and that students take responsibility for their own learning - thus not requiring further accountability from CTFs. What students instead find most useful is mentorship and supportiveness.

*“Administrative staff may see CTFs similar to lecturers- a bit of an impersonal relationship and maybe not impacting on your placement experience that much. At [University] during clinical years there is no other staff that get to know you other than CTFs really so in the past they have been a great source of personal academic and emotional support. Because of this, personal qualities are important.” - Year 5, respondent 36*

Perceived differences regarding specialist knowledge requirements were also noted by students.

*“I think as medical students it is helpful for us to have CTFs closer to our knowledge level and who are able to understand our position more. This would mean FY2+ is the ideal time to be a CTF. I wonder if admin staff and faculty feel that more specialised training is needed but really from a student perspective we just want and need someone who can guide us through medical school.” - Year 5, respondent 38*

A common theme from students was that they did not know if a disparity existed between students and admin/faculty, and a lack of open communication between the two groups often led to assumptions that a disparity did exist. Two key reasons were mentioned to explain this. Firstly, that students have encountered CTFs of varying quality in the past and therefore assume there must be a lack of standardisation in the recruitment of CTFs by admin/faculty members. Secondly, students stated that admin/faculty lack the understanding of the experience of being on clinical placement as a medical student.

*“I have no idea how CTFs train to become CTFs, so I won't know what the undergrad/admin staff think is a good CTF. However judging by the disparity of quality of ctfs we perceive as medical students, which then implies poor standardisation of CTF quality through the system, I would say I agree that what students are looking for is different to what the system thinks makes a good CTF.” - Year 3, respondent 33*

*“Yes, I think med students and CTFs probably know what qualities work and at the end of the day that's what matters … For example, I feel as though the administrative team have no idea what it is like to be on placement as a student, where as CTFs do, therefore I think they are the ones in the position to determine what sort of CTFs should be teaching in the future and how teaching should be set out in the hospital setting.” - Year 3, respondent 30*

**CTFs vs admin/faculty**

CTFs also noted some disparities they believed existed between themselves and admin/faculty, some of which overlapped with students’ beliefs. These include the preference for early career over specialist knowledge, and pastoral support over a purely organisational and lecturing role. CTFs believed admin/faculty place a higher value on professionalism, punctuality, and strictness - whilst they instead prioritise approachability, friendliness, and enthusiasm.

*“I expect that the admin staff value professional, punctual and perhaps stricter teachers where CTFs value approachability, friendliness, and enthusiasm. There may also be a disparity between the expectations of knowledge (e.g. faculty expect CTFs to “know” more)” - CTF, respondent 4*

**Students vs CTFs**

Students and CTFs largely agreed on what makes a good CTF, potentially attributed to their shared experiences of life as a medical student on placement. However, two students noted disparities between themselves and some CTFs who they have encountered. One student stated that some CTFs “have not really cared about teaching us and cared more about the qualification/application points they were getting”. Another student acknowledged that different CTFs have different teaching styles, perhaps shaped from previous experience, meaning that medical students who have a preference for a particular style will never have their wishes met all the time.
